# Saddle Nose Deformity Reconstruction in Yemen: A Prospective Study of the Diced Cartilage Fascia Technique

**DOI:** 10.7759/cureus.79319

**Published:** 2025-02-19

**Authors:** Yahia A Alsiaghi, Mohammed H Al-Shoaibi, Mohaned Y Al-ajaly, Ayman M Ghanem, Haitham M Jowah

**Affiliations:** 1 Department of Plastic Surgery, Typical Police Hospital, Sana’a, YEM; 2 Department of Plastic Surgery, General Military Hospital, Sana'a, YEM; 3 Department of Plastic Surgery, Elite Hospital, Sana'a, YEM; 4 Department of Surgery, Faculty of Medicine and Health Sciences, Sana’a University, Sana’a, YEM; 5 Department of Surgery, 21 September University of Medical and Applied Sciences, Sana'a, YEM

**Keywords:** diced cartilage fascia technique, nasal reconstruction, resource-limited settings, rhinoplasty, saddle nose deformity

## Abstract

Background: Saddle nose deformity presents significant challenges, particularly in conflict-affected and resource-limited settings. The diced cartilage fascia (DCF) technique, which involves wrapping diced cartilage in fascia, has shown potential for addressing these challenges but remains underexplored. This study aimed to evaluate the efficacy, safety, and patient satisfaction of the DCF technique for saddle nose deformity reconstruction in Yemen.

Methods: This was a prospective study that included 30 patients who underwent saddle nose reconstruction using the DCF technique between January 2020 and January 2023. Data collected included patient demographics, preoperative and postoperative assessments using the Nasal Obstruction Symptom Evaluation (NOSE) and Rhinoplasty Outcome Evaluation (ROE) scores, A-B line measurements, and complication rates. Wilcoxon signed-rank test was used to compare preoperative and postoperative outcomes.

Results: The study included 30 patients, 12 male (40%) and 18 female (60%), with a mean age of 28.5 years. Significant improvements were observed, with the mean A-B line measurement increasing from 16.75 mm to 21 mm (p < 0.001) and the mean NOSE score improving from 52.8 ± 22.2 to 5 ± 5.1 (p < 0.01). Patient satisfaction was high, with 29 patients (96.7%) reporting excellent outcomes. Postoperative complications occurred in two patients (6.6%). Nasal deviation was noted in one patient (3.3%) and was managed with reoperation, while infection in one patient (3.3%) was treated conservatively.

Conclusions: The DCF technique is a safe, effective, and promising method for reconstructing saddle nose deformities in resource-limited settings. It yields significant functional and aesthetic improvements with high patient satisfaction and minimal complications. Larger studies with extended follow-up are recommended to confirm these findings.

## Introduction

Nasal deformities, particularly dorsal nasal deformities such as saddle noses, are among the most challenging problems in rhinoplasty, significantly impacting both facial aesthetics and nasal function. This condition results in a characteristic "saddle-like" appearance and can arise from various causes, including trauma, congenital abnormalities, infections, autoimmune diseases, and iatrogenic factors [1,2]. Traditional treatment methods, including bone grafts, solid cartilage grafts, and dermofat grafts, have limitations such as rapid degradation, graft displacement, and failure to restore the nose's original shape. Additionally, the use of cadaveric and artificial grafts is often constrained by limited availability, high costs, the risk of disease transmission, and potential foreign body reactions [3,4].

One promising surgical approach for correcting saddle nose deformity is the diced cartilage fascia (DCF) technique. This method involves harvesting the patient’s cartilage, typically from the nasal septum or ear, dicing it into small pieces, and wrapping it in a fascia layer, usually harvested from the temporal or deep fascia [4,5]. The resulting graft is malleable, allowing for precise augmentation and reshaping of the nasal dorsum, with the fascia layer facilitating integration into the recipient site. The DCF technique offers several advantages over traditional methods, including the ability to customize the shape and volume of the graft, reduced risk of graft resorption or displacement, improved contouring and blending with surrounding nasal structures, and potential for improved long-term stability and aesthetic outcomes [6,7].

In resource-limited settings like Yemen, managing complex nasal deformities presents significant challenges. High costs, limited material availability, and the burden of conflict further complicate the situation. Therefore, alternative techniques that can effectively address nasal deformities while being feasible in such environments are urgently needed.

This study aimed to evaluate the efficacy, safety, and patient satisfaction of the DCF technique for saddle nose deformity reconstruction in Yemen. By focusing on a conflict-affected, resource-limited setting, we investigated the technique’s outcomes and compared them with those of more stable environments. This prospective study provides valuable insights for surgeons working in similar settings.

This article was previously posted to the Research Square preprint server on August 7, 2024 [8].

## Materials and methods

Study design and setting

This prospective case series study was conducted at three plastic and reconstructive surgery departments in Sana’a, Yemen: Military General Hospital, Typical Police Hospital, and Elite Hospital. The study was conducted over three years, from January 2020 to January 2023. All surgical procedures were performed by a single plastic surgeon team to ensure consistency in technique and standardization of the diced cartilage fascia (DCF) grafting process.

Patient selection

A total of 30 consecutive patients with saddle nose deformity were included in the study. Inclusion criteria were clinically and radiologically confirmed saddle nose deformity, significant nasal obstruction or breathing difficulties, esthetic concerns regarding nasal appearance, age above 18 years, and willingness to cease smoking before and after surgery. Exclusion criteria included patients younger than 18 years (unless the deformity severely impacted the quality of life), severe comorbid conditions posing high surgical risks, psychological instability (including body dysmorphic disorder), active infections, known allergies to grafting materials, or pregnancy.

Surgical technique

Preoperative Assessment and Planning

Preoperative planning included a consultation to assess nasal anatomy and define aesthetic goals using computer imaging. Patients were marked in the upright position. If costal cartilage harvesting was planned, the inframammary or infrapectoral crease and xiphoid were marked.

Intraoperative Steps

**Figure 1:**
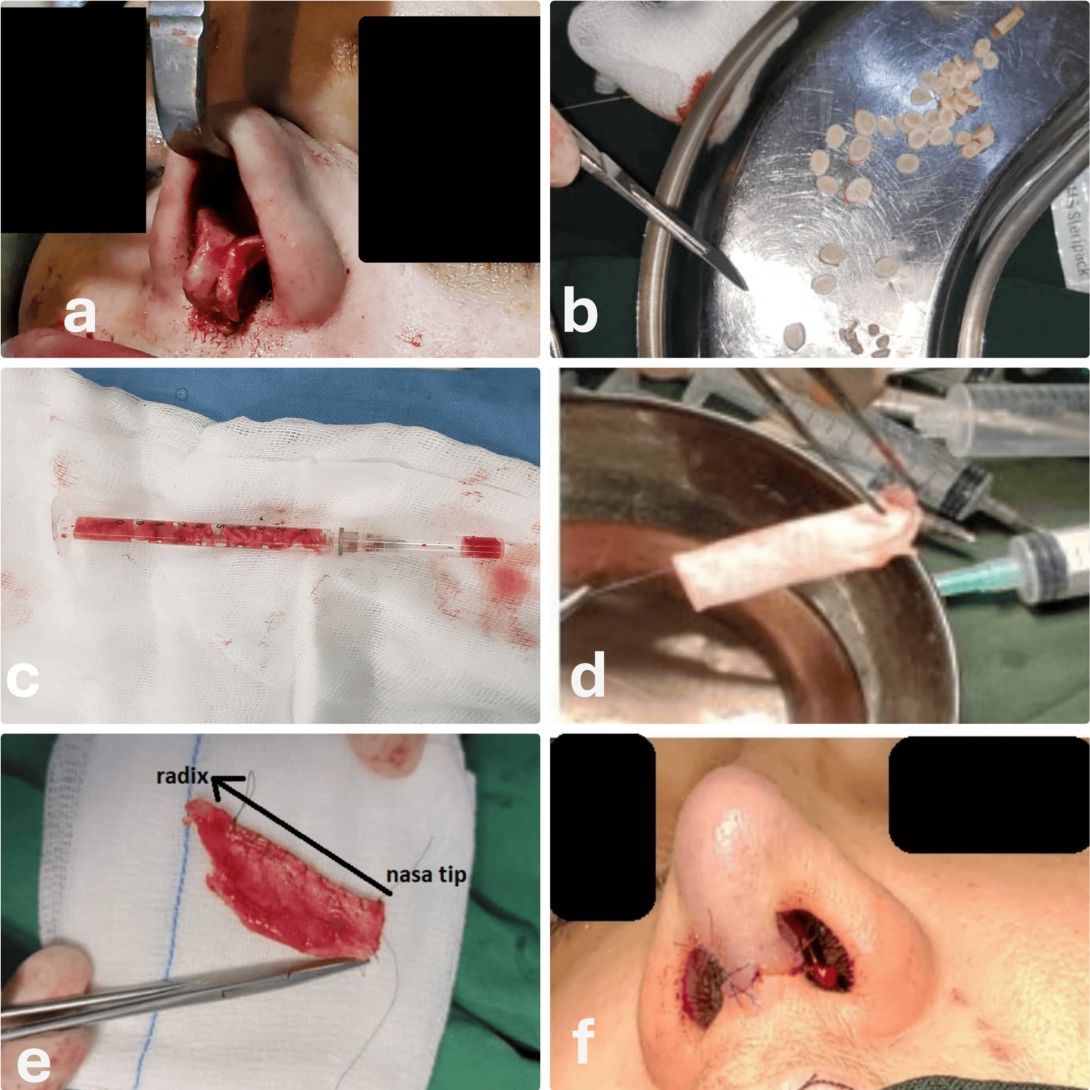
The surgical procedure steps for nasal augmentation using diced cartilage fascia technique Sequential steps include: (a) Open rhinoplasty approach for access to the nasal framework; (b) Dicing and preparation of graft materials; (c) Packing diced cartilage into a syringe; (d) Harvesting of temporal or deep fascia; (e) Wrapping fascia around a catheter to form a sheath; (f) Closure of the nasal incision and application of a septal splint.

Under general anesthesia with local infiltration of local anesthetics, an open rhinoplasty approach was employed to expose the nasal framework. Harvested graft materials, including costal cartilage, septal cartilage, conchal cartilage, and cartilage trimmed from the lateral crura, were employed. The cartilage was diced into 0.5-1 mm cubes using a No. 11 blade and packed into a 1 cc syringe. The fascia was primarily harvested from the tensor fascia lata (TFL) or, in some cases, from the temporal region. A rectangular piece measuring 40 x 20 mm was obtained. The fascia was wrapped around a 1 mm diameter catheter and secured with 5-0 polydioxanone (PDS) sutures to create a hollow, sleeve-like sheath. The diced cartilage was then inserted into the fascial cylinder to create a malleable graft (Figure 1).

The graft was positioned and shaped to achieve the desired aesthetic outcome, and corset sutures were used for stability. Fenestration was added to facilitate vascular integration. The nasal skin was redraped, and the incision was closed. A septal splint was applied if the septal cartilage was used.

Postoperative Steps

Postoperative care included the application of a thermoplastic splint to maintain the graft shape. The splint was set using ice-cold water and removed after one week, along with any percutaneous sutures.

Data collection

Demographic and clinical data were prospectively collected, including patient age, sex, and deformity etiology. Preoperative and postoperative assessments included validated Arabic versions of the Nasal Obstruction Symptom Evaluation (NOSE) and Rhinoplasty Outcome Evaluation (ROE) scores [9], A-B line measurements, and standardized photographs. Surgical details such as graft harvest site, volume, and complications were recorded. Patients were followed up at monthly intervals for six months. All data were securely stored and anonymized to ensure confidentiality.

Outcome measures and assessment

**Figure 2:**
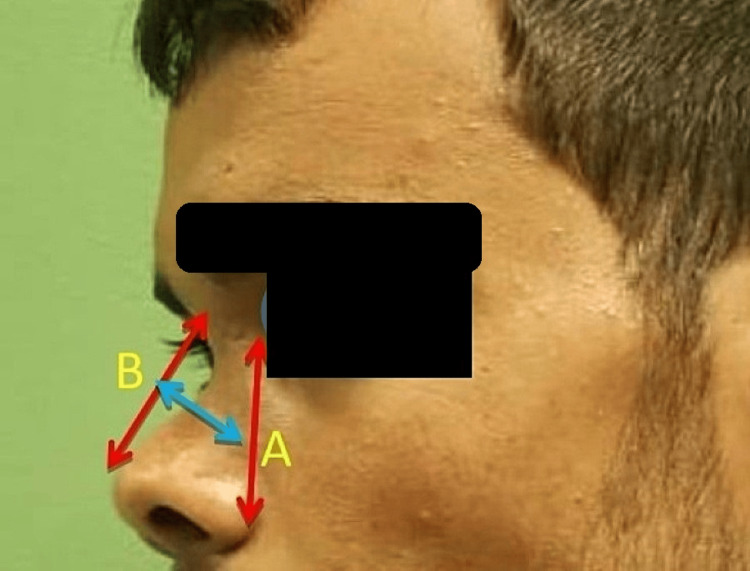
Outcome assessment of nasal height changes using the A-B line measurement The A-B line was used to quantify changes in nasal height, defined as the difference in distance (measured in millimeters) between Point A and Point B before and after surgery. Point A is the midpoint between the inner canthus and the alar base, while Point B represents the midpoint of the line from the nasal bridge to the supratip. This method provides a standardized evaluation of postoperative nasal height changes.

The primary outcomes were improvements in nasal esthetics and function, assessed using validated Arabic versions of the NOSE and ROE scores [9]. Secondary outcomes included postoperative complications such as infection, recurrence, and visible deformities, as well as patient satisfaction. Objective assessments included preoperative and postoperative A-B line measurements and standardized photographs. The A-B line is defined with Point A as the midpoint of the line at the nasal base, located between the inner canthus and alar base, and Point B as the midpoint along the nasal dorsum, extending from the radix to the supratip (Figure 2).

The photographic assessments were compared with baseline images. The complications were documented and managed appropriately.

Data analysis

Data analysis was performed using IBM SPSS Statistics for Windows, Version 26.0 (2019; IBM Corp., Armonk, New York, United States). Descriptive statistics included means, medians, interquartile ranges (IQR), standard deviations (SDs), frequencies, and percentages. Data normality was tested using the Shapiro-Wilk test. Comparative analyses of preoperative and postoperative variables were conducted using the Wilcoxon signed-rank test for nonparametric paired data, including changes in NOSE and ROE scores and A-B line measurements. A p-value <0.05 was considered statistically significant. Effect sizes were calculated to determine the magnitude of observed changes.

Ethics approval and consent for publication

The study was approved by the Institutional Review Board of the Yemeni Board for Medical Specialisation, Sana’a, Yemen (approval number: IRB/YBMS/2019/047, issued December 15, 2019). All patients provided written informed consent for participation and publication of their data and images. The study adhered to the ethical standards outlined in the Declaration of Helsinki and subsequent amendments.

## Results

Demographic characteristics

The study included 30 participants, 12 men (40%) and 18 women (60%). Most participants (n = 17, 56.7%) were aged 18-29 years, followed by 12 participants (40%) in the age group of 30-49 years, and one participant (n = 1, 3.3%) aged 50 years or older. The majority of nasal deformities were post-traumatic (n = 19, 63.3%), whereas congenital deformities accounted for 11 participants (36.7%). The median duration of nasal deformity was 5.5 years (range: 1-40 years) (Table 1).

**Table 1:** Demographic characteristics of the study population (N=30) IQR: interquartile range

Demographic characteristics	Values
Sex, n (%)	
Female	18 (60%)
Male	12 (40%)
Age group (years), n (%)	
18-29	17 (56.7%)
30-49	12 (40%)
≥ 50	1 (3.3%)
Age (years), median (IQR)	28.5 (19-50)
Aetiology of the deformity, n (%)	
Post-traumatic	19 (63.3%)
Congenital	11 (36.7%)
Duration of nasal deformity (years), median (IQR)	5.5 (1-40)

Preoperative assessment

Before the surgery, 43.3% of participants (n = 13) had moderate NOSE scores, 26.7% (n = 8) had severe NOSE scores, 20% (n = 6) had extreme NOSE scores, and 10% (n = 3) had mild NOSE scores. The mean NOSE score was 52.8 ± 22.2, with a median score of 50 (range: 25-95). The mean preoperative A-B line length was 16.75 mm (range: 16-18 mm). The mean volume of diced cartilage injected during surgery was 1.2 ml (range: 1-2 ml). ROE scores categorized participants as follows: 40% (n = 12) acceptable, 40% (n = 12) good, 13.3% (n = 4) excellent, and 6.7% (n = 2) poor, with a mean score of 50 ± 19.5 and a median score of 52 (range: 21-79) (Table 2).

**Table 2:** Preoperative assessment of the study participants (N=30) Categorical variables are expressed as frequencies and percentages; continuous variables are presented as median (IQR) or mean ± SD based on normality distribution NOSE: Nasal Obstruction Symptom Evaluation; ROE: Rhinoplasty Outcome Evaluation; IQR: interquartile range

Preoperative variables	Values
The NOSE score category, n (%)	
Mild (5 < 25)	3 (10%)
Moderate (30 < 50)	13 (43.3%)
Severe (55 < 75)	8 (26.7%)
Extreme (80-100)	6 (20.0%)
Overall NOSE score	
Mean ± SD	52.8 ± 22.2
Median (IQR)	50 (25-95)
A-B line (mm), median (IQR)	16.75 (16-18)
Amount of diced cartilage injected (ml), , median (IQR)	1.2 (1-2)
ROE score categories, n (%)	
Poor (0 < 25)	2 (6.7%)
Acceptable (25 < 50)	12 (40.0%)
Good (50 < 75)	12 (40.0%)
Excellent satisfaction (75-100)	4 (13.3%)
Overall ROE score	
Mean ± SD	50 ± 19.5
Median (IQR)	52 (21-79)

Postoperative complications

Postoperative complications were observed in 6.6% (n = 2) of the participants. Specifically, 3.3% (n = 1) of the participants experienced nasal deviation, which was corrected with reoperation. Another 3.3% (n = 1) developed an infection, which was managed conservatively (Table 3).

**Table 3:** Postoperative complications and management strategies (N=2)

Variable	Frequency	Percentage	Management
Postoperative complications	2	6.6	-
Nasal deviation	1	3.3	Reoperation and nasal correction
Infection	1	3.3	Conservative management

Postoperative outcome assessment

After surgery, the mean NOSE score significantly decreased to 5 ± 5.1. The median A-B line length increased to 21 mm (range: 20-23 mm), and the mean dorsal height gain was 4.56 mm (range: 4-6 mm). ROE scores indicated that 96.7% (n = 29) of participants reported excellent satisfaction, whereas 3.3% (n = 1) reported good satisfaction. The mean ROE score was 93 ± 7.93, with a median score of 95.8 (range: 67-100) (Table 4).

**Table 4:** Postoperative outcomes of the participants (N = 30) Categorical variables were analyzed by frequency and percentages; continuous variables were expressed by Median (IQR) or Mean ± SD based on normality distribution. NOSE: Nasal Obstruction Symptom Evaluation; ROE: Rhinoplasty Outcome Evaluation; IQR: interquartile range

Postoperative variable	Values
NOSE score category, n (%)	
Mild (5 < 25)	30 (100%)
Overall NOSE score	
Mean ± SD	5 ± 5.1
Median (IQR)	5 (0-18)
A-B line (mm), median (IQR)	21 (20-23)
Gained dorsal height (mm), median (IQR)	4.56 (4-6)
ROE score categories, n (%)	
Good (50 < 75)	1 (3.3%)
Excellent satisfaction (75-100)	29 (96.7%)
Overall ROE score	
Mean ± SD	93 ± 7.93
Median (IQR)	95.8 (67-100)

Comparison of preoperative and postoperative outcomes

Significant improvements were observed in preoperative versus postoperative outcomes. The A-B line length increased from 16.75 mm (IQR: 16-18 mm) preoperatively to 21 mm (IQR: 20-23 mm) postoperatively (p = 0.002). The mean NOSE score decreased significantly from 52.8 ± 22.2 to 5 ± 5.1 (p = 0.044), indicating reduced nasal obstruction symptoms. Additionally, the mean ROE score increased from 50 ± 19.5 preoperatively to 93 ± 7.93 postoperatively (p = 0.001), reflecting significantly higher patient satisfaction (Table 5).

**Table 5:** Comparison of preoperative and postoperative NOSE and ROE scores and A-B line length *Significant p-value < 0.05 using the Wilcoxon signed-rank test for preoperative and postoperative comparisons NOSE: Nasal Obstruction Symptom Evaluation; ROE: Rhinoplasty Outcome Evaluation; IQR: interquartile range

Variables	Preoperative values	Postoperative values	P-value
A-B line (median, IQR)	16.75 (16-18)	21 (20-23)	0.002*
Total NOSE score (mean ± SD)	52.8 ± 22.2	5 ± 5.1	0.044*
Total ROE score (mean ± SD)	50 ± 19.5	93 ± 7.93	0.001*

Presentation of some cases

Case One

**Figure 3:**
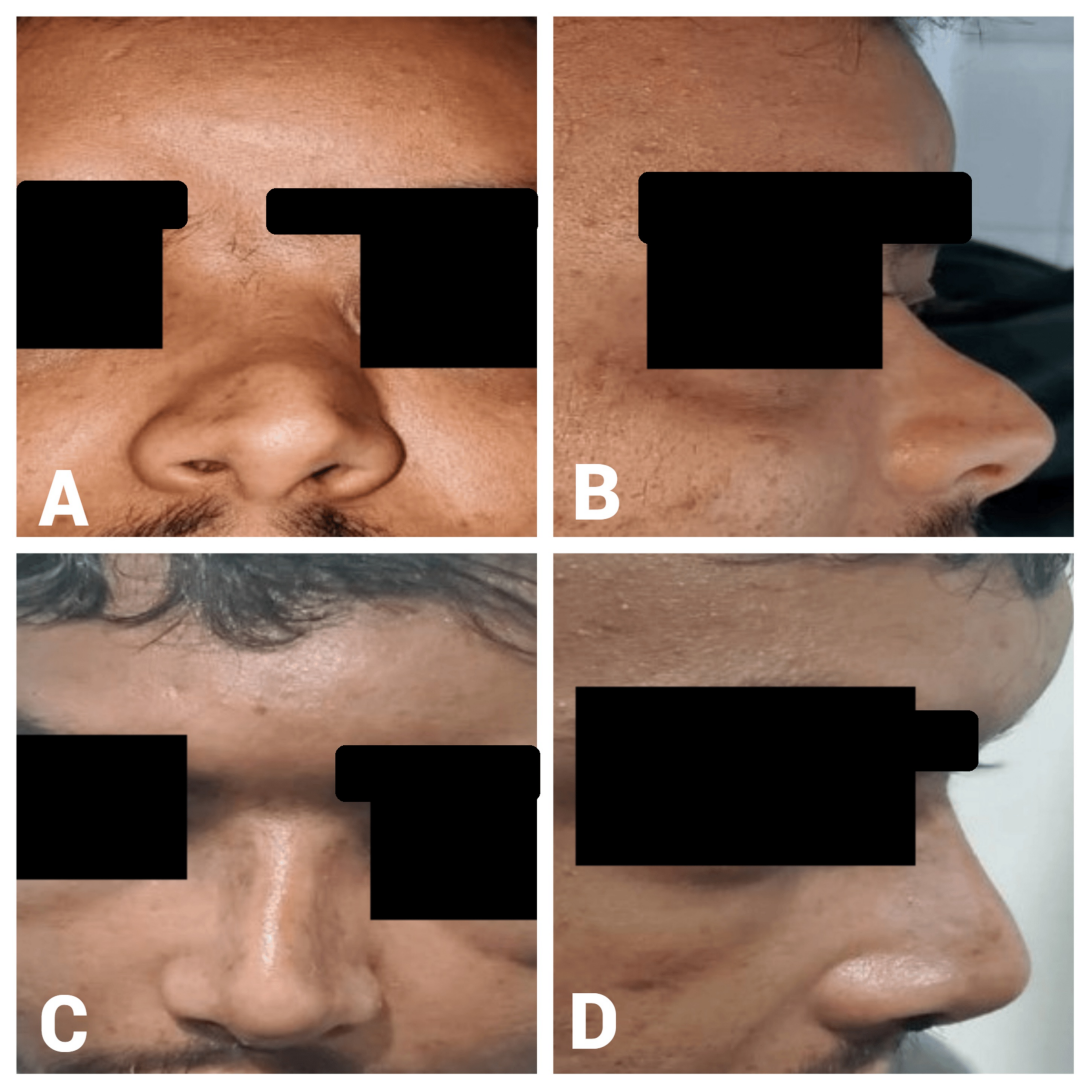
Case 1. A 22-year-old male patient with a one-year history of post-traumatic nasal obstruction and dorsal deformity who underwent DCF reconstruction. Preoperative frontal view (A) demonstrates a depressed nasal dorsum and asymmetrical alar structure caused by post-traumatic nasal deformity, while the lateral view (B) highlights a flat nasal dorsum with deficient dorsal projection. Postoperative frontal view (C) shows restored nasal symmetry and enhanced dorsal contour following diced cartilage fascia reconstruction, and the lateral view (D) illustrates improved dorsal projection and refined nasal tip contour. DCF: diced cartilage fascia

A 22-year-old male presented with post-traumatic nasal obstruction, dorsal nasal deformity (osseocartilaginous), and alar asymmetry for one year. The preoperative A-B line length was 17 mm. Intraoperatively, 2 ml of diced cartilage was injected. The postoperative A-B line measurement increased to 22 mm, resulting in a 5-mm gain in dorsal height (Figure 3).

Case Two

**Figure 4:**
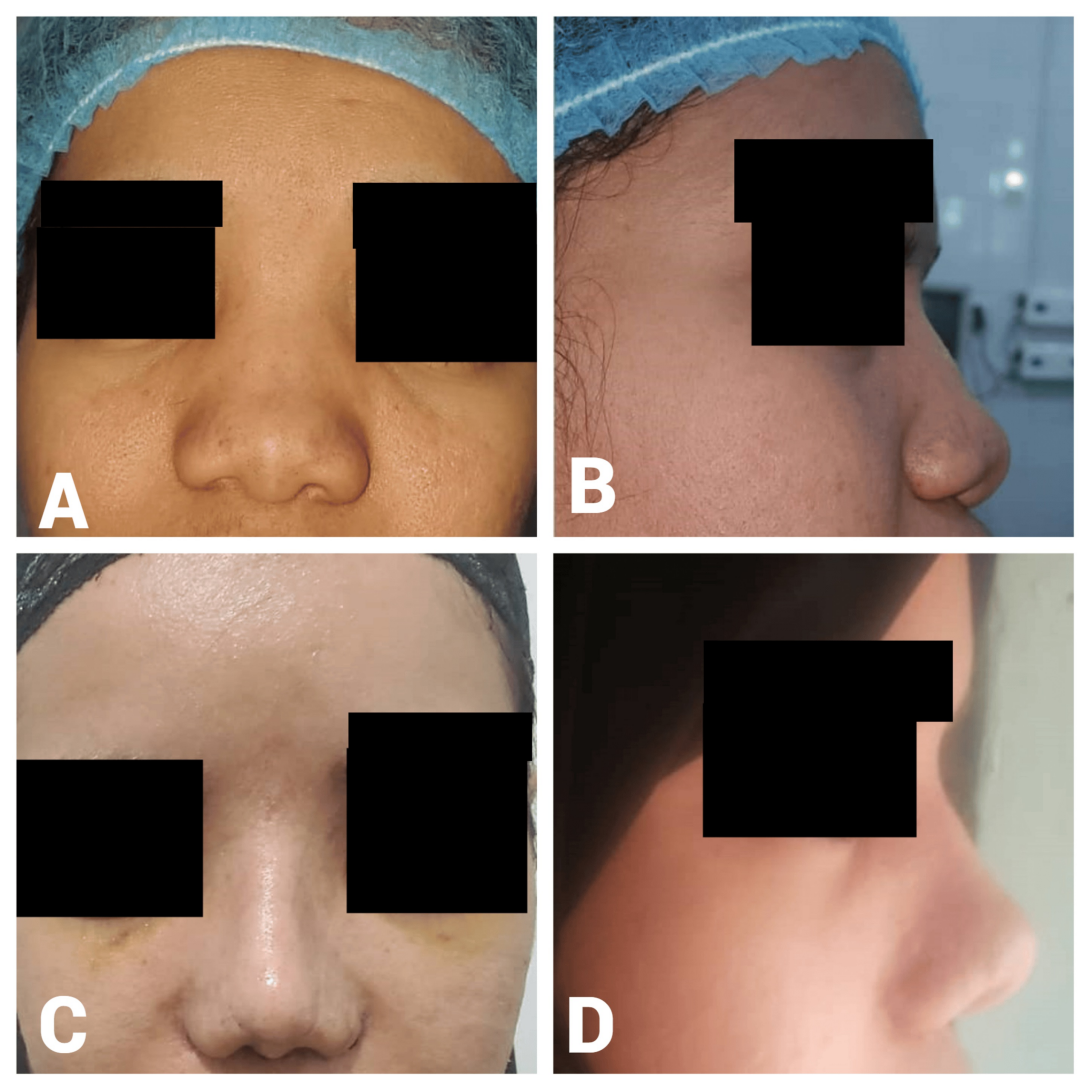
Case 2. A 27-year-old female patient who presented with a one-year history of post-traumatic nasal deformity characterized by a flat nasal dorsum and drooping nasal tip. Preoperative frontal view (A) shows a flat nasal dorsum and wide nasal tip resulting from post-traumatic deformity, while the lateral view (B) highlights a depressed nasal dorsum and insufficient nasal projection. Postoperative frontal view (C) demonstrates improved nasal symmetry and contour, and the lateral view (D) illustrates increased dorsal height and refined nasal tip projection.

A 27-year-old female presented with a one-year postnasal trauma with a flat nasal dorsum, droopy-wide nasal tip, and short columella. The preoperative A-B line length was 17 mm. Intraoperatively, 1 ml of diced cartilage was used. The postoperative A-B line measurement increased to 21 mm, resulting in a 4-mm gain in the dorsal height (Figure 4).

Case Three

**Figure 5:**
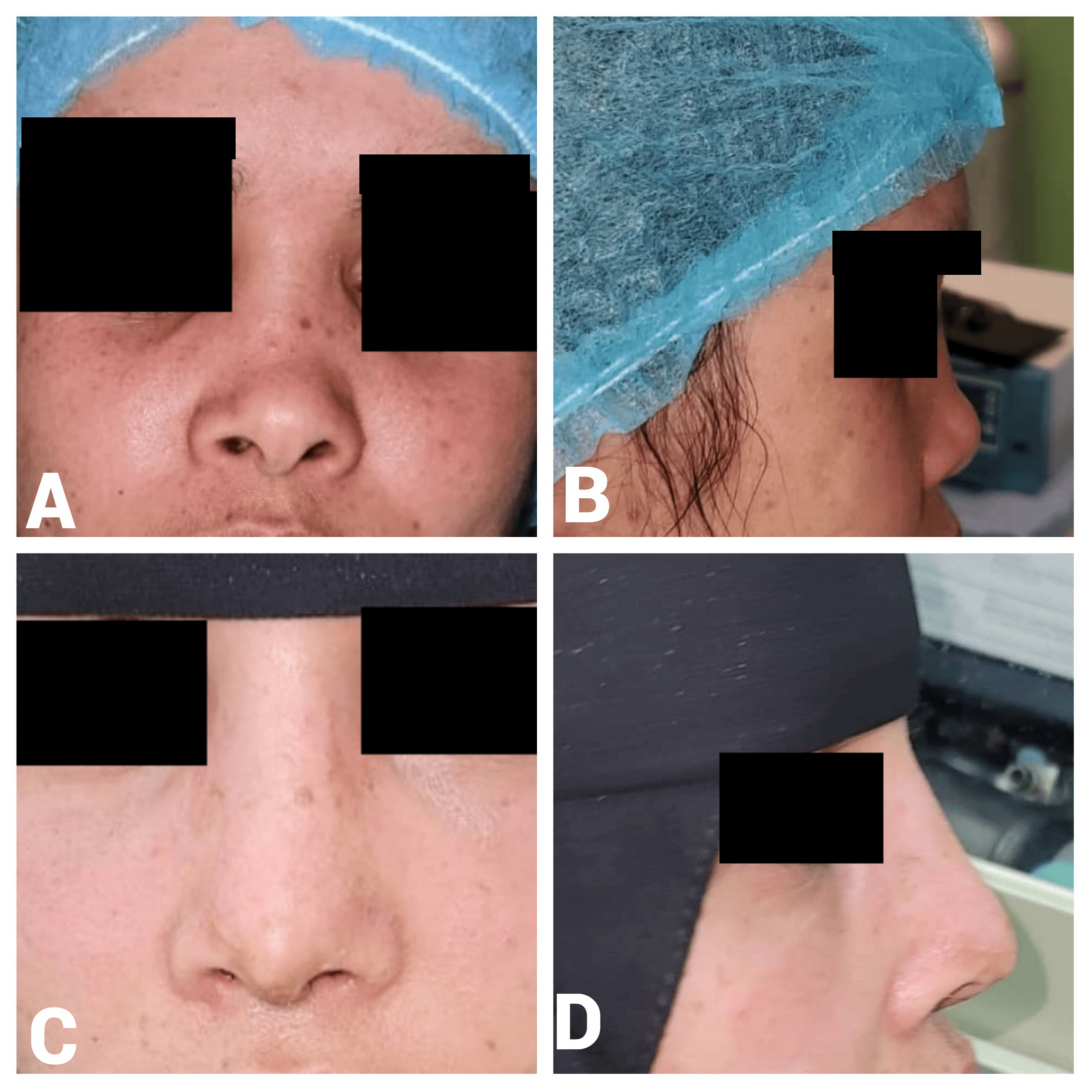
Case 3. A 36-year-old female who presented two years post trauma with a saddle-shaped nasal deformity, drooping nasal tip, and alar retraction. Preoperative frontal view (A) displays a saddle-shaped nose with a drooping nasal tip and alar retraction, while the lateral view (B) indicates a depressed nasal dorsum and inadequate nasal projection. Postoperative frontal view (C) shows corrected nasal symmetry and improved contour, and the lateral view (D) demonstrates enhanced dorsal projection and tip elevation.

A 36-year-old female patient presented two years after nasal trauma with a saddle-shaped nose, drooping nasal tip, alar retraction, and small nose. The preoperative A-B line measurement was 16 mm. Intraoperatively, 1 ml of diced cartilage was used. The postoperative A-B line measurement increased to 21 mm, resulting in a 5-mm gain in dorsal height (Figure 5).

Case Four

**Figure 6:**
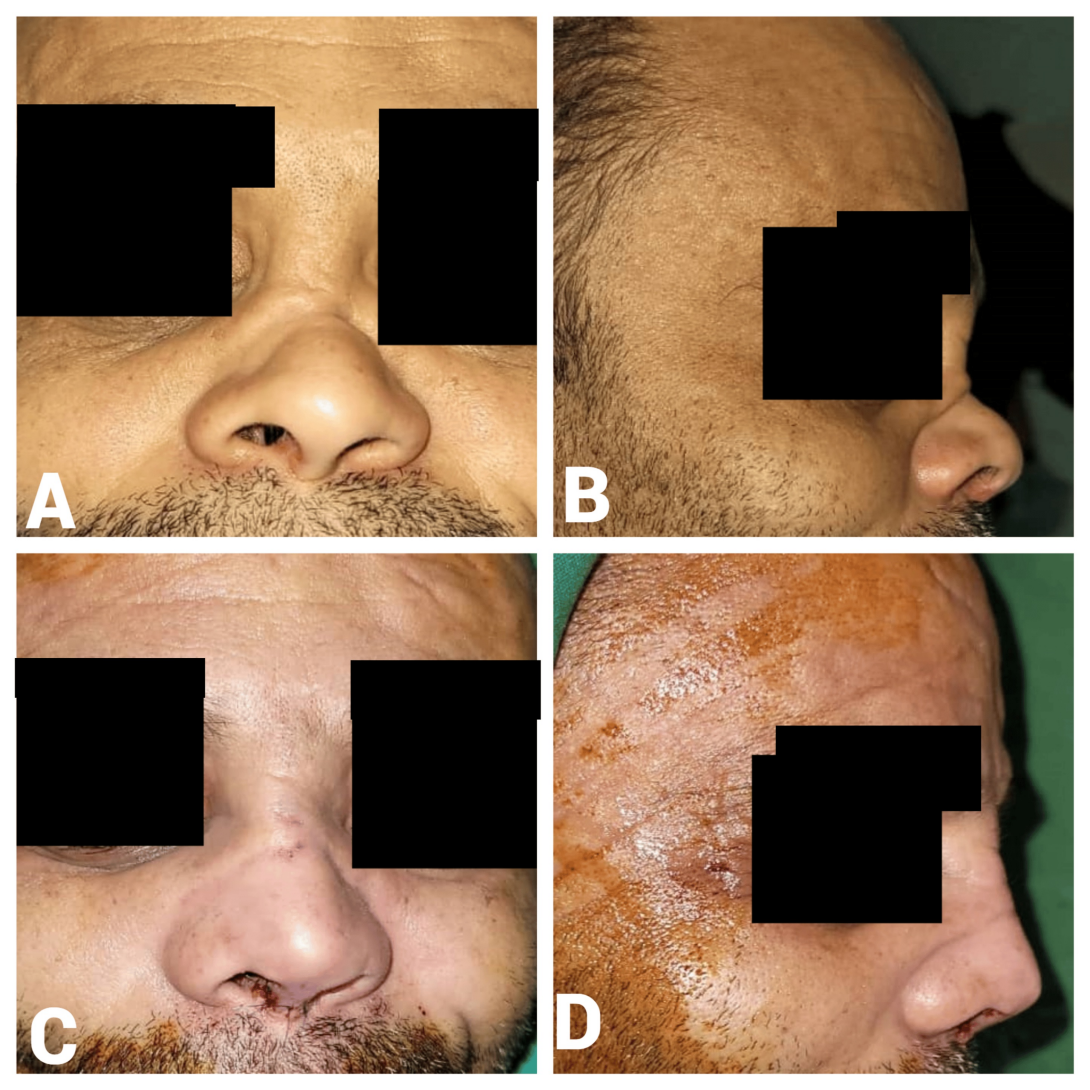
Case 4. A 40-year-old male patient who presented with post-traumatic nasal obstruction and dorsal nasal collapse. Preoperative frontal view (A) shows dorsal nasal collapse and a wide nasal tip, while the lateral view (B) indicates a depressed nasal dorsum and inadequate projection. Postoperative frontal view (C) demonstrates corrected nasal structure and improved symmetry, and the lateral view (D) displays enhanced dorsal height and improved nasal tip projection.

A 40-year-old male patient presented with post-traumatic nasal obstruction, dorsal nasal collapse (osseocartilaginous), and a wide nasal tip. The preoperative A-B line length was 17 mm. Intraoperatively, 2 ml of diced cartilage was used. The postoperative A-B line measurement increased to 22 mm, resulting in a 5-mm gain in dorsal height (Figure 6).

Case Five

**Figure 7:**
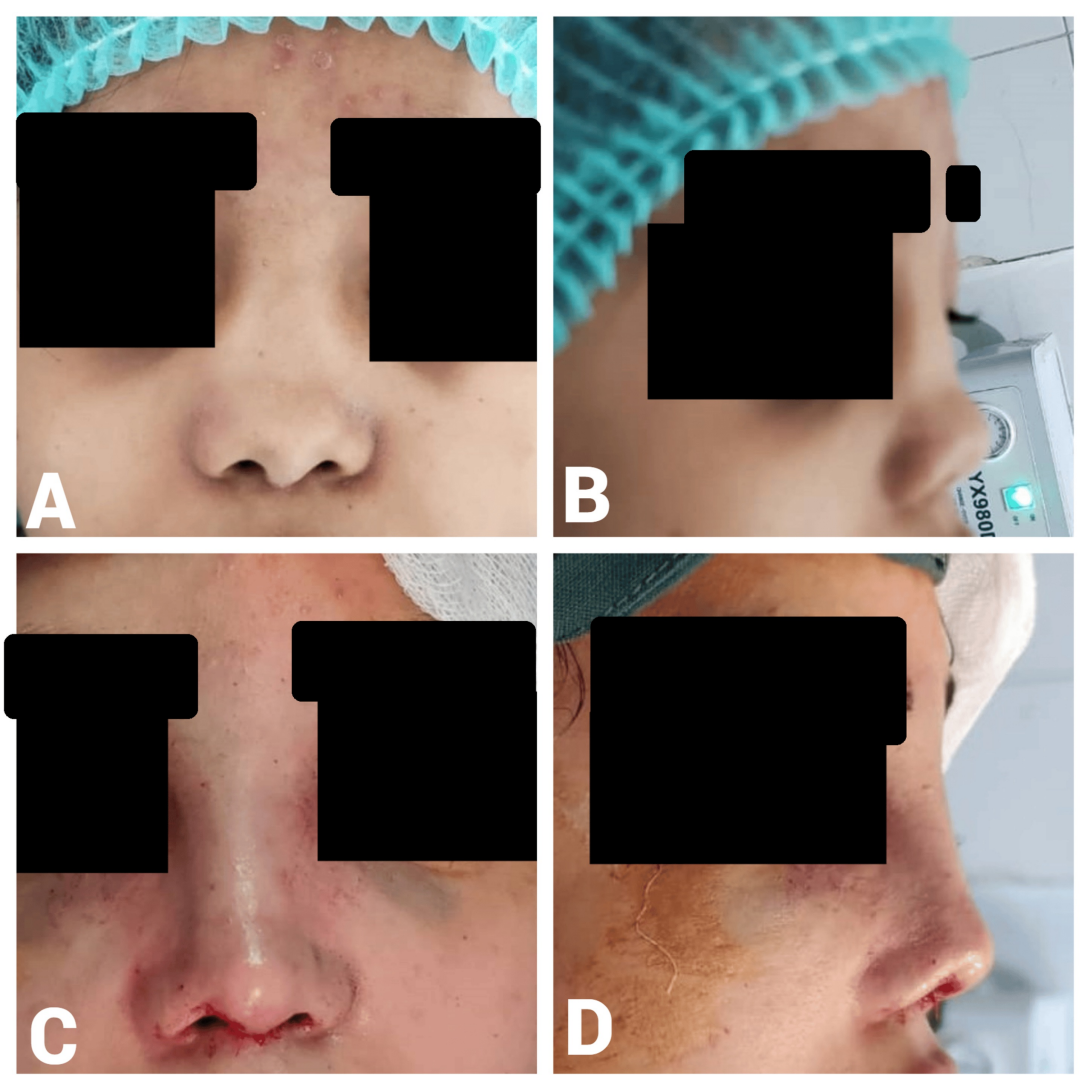
Case 5. A 20-year-old female with a four-year history of postnasal trauma who presented with a saddle-shaped nose and previous septoplasty. Preoperative frontal view (D) shows a depressed nasal dorsum and asymmetry, while the lateral view (A) demonstrates a saddle-shaped nose with inadequate nasal projection and columella. Postoperative frontal view (C) demonstrates restored nasal symmetry and improved esthetics, and the lateral view (B) indicates enhanced dorsal nasal projection and improved nasal contour.

A 20-year-old female patient presented with a four-year postnasal trauma with a saddle-shaped nose and short nose. She had a history of one previous septoplasty. The preoperative A-B line measurement was 16 mm. Intraoperatively, 1 ml of diced cartilage was used. The postoperative A-B line measurement increased to 21 mm, resulting in a 5-mm gain in dorsal height (Figure 7).

## Discussion

The findings of this study highlight the significant benefits and feasibility of the DCF technique for rhinoplasty, particularly in resource-limited and conflict-affected settings. The demographic distribution of participants, predominantly young adults with a higher percentage of female patients, aligns with existing studies indicating that younger populations often seek rhinoplasty to address functional and aesthetic concerns [10].

Our study demonstrated substantial improvements in nasal aesthetics and function, as evidenced by the increase in A-B line measurements from a preoperative mean of 16.75 mm to a postoperative mean of 21 mm. These results are consistent with prior research showing the efficacy of the DCF technique in enhancing nasal projection and contour [6,7]. The significant reduction in NOSE scores from 52.8 ± 22.2 to 5 ± 5.1 further supports the dual functional and aesthetic advantages of rhinoplasty [11-13].

High levels of patient satisfaction, with 96.7% reporting excellent outcomes, underscore the success of DCF. This finding is corroborated by the increase in ROE scores from 50 ± 19.5 to 93 ± 7.93, indicating significant enhancements in patients’ quality of life. These findings are consistent with other studies showing substantial improvements in patient-reported outcomes using tools such as ROE and FACE-Q [14-16]. Additionally, younger and female patients reported higher satisfaction levels, which is consistent with previous studies [15,17].

The complication rate of 7% with manageable issues such as nasal deviation and infection is consistent with the existing literature on DCF safety. Another study by Li et al. reports complication rates for DCF grafts, including infection and visible irregularities, as 11.5%, demonstrating the relative safety of the technique [5]. Compared with alternative grafting methods, DCF offers lower or comparable complication rates and reduced risks of graft resorption and displacement [18-20].

The prevalence of post-traumatic nasal deformities in 63.3% of cases emphasizes the impact of trauma on nasal structure, consistent with prior findings that up to 50% of nasal fracture patients may require reconstructive surgery [21,22]. The high satisfaction rates in post-traumatic rhinoplasty, as reported in this study, are in agreement with existing research demonstrating success in addressing trauma-induced nasal deformities [23,24].

The versatility of DCF in increasing dorsal projection and addressing cases with depleted donor sites or poor-quality cartilage highlights its adaptability for complex scenarios [19]. The cost-effectiveness of this method and its dependence on locally sourced cartilage make it highly suitable for resource-constrained settings, reducing the need for expensive surgical materials while maintaining efficacy [19,25].

While advancements such as three-dimensional imaging and virtual planning have enhanced rhinoplasty outcomes, the fundamental principles of DCF remain effective even in the absence of such technologies [26]. Additional reconstructive options, such as microsurgical free flaps and dermal regeneration templates, offer alternatives but may not be as practical in low-resource settings [27].

Challenges and opportunities in performing rhinoplasty in resource-limited settings have been explored. For instance, cleft lip repair combined with primary rhinoplasty under local anesthesia has proven effective in resource-poor environments [28]. Single-stage reconstruction techniques tailored to patient preferences further highlight the importance of adaptable approaches [28,29]. This study contributes to the growing body of evidence and demonstrates that DCF is a practical, minimally invasive option for addressing saddle nose deformity with minimal donor site morbidity.

Limitations

Despite these results, this study has limitations. Although the study was conducted across multiple centers, all surgeries were performed by a single plastic surgeon team, which may limit the generalizability of the findings to other surgical teams with varying levels of expertise. While this approach ensures consistency in surgical technique, it may not fully represent outcomes achievable by a broader range of surgeons. Additionally, the sample size remains relatively small, and future research should include larger, more diverse populations to validate these results. Longer follow-up periods are necessary to assess the durability and potential long-term complications of DCF. The absence of a control group and lack of cost-effectiveness analysis are additional limitations that should be addressed in future studies. Furthermore, potential confounding factors such as patient selection and postoperative care may have influenced the outcomes. While we used validated outcome measures to enhance the reliability of our findings, future randomized controlled trials involving multiple surgical teams are needed to further establish the efficacy and safety of the DCF technique.

## Conclusions

The DCF technique is an effective, safe, and feasible option for rhinoplasty in resource-limited settings, providing significant improvements in nasal aesthetics and function, high patient satisfaction, and a low complication rate. However, the observational nature of this study, the involvement of a single plastic surgeon team, and the potential influence of confounding factors should be acknowledged. While the single-surgeon team ensured consistency in surgical technique, it may limit the generalizability of the findings to other surgical teams. Future studies with larger sample sizes, diverse populations, extended follow-ups, and involvement of multiple surgical teams are necessary to further establish the effectiveness of this approach and to inform clinical decision-making.
